# Fluctuation of photon-releasing with ligand coordination in polyacrylonitrile-based electrospun nanofibers

**DOI:** 10.1038/s41598-020-57641-3

**Published:** 2020-01-22

**Authors:** Zhimin Yu, Lifan Shen, Desheng Li, Edwin Yue Bun Pun, Xin Zhao, Hai Lin

**Affiliations:** 1 School of Information Science and Engineering, Dalian Polytechnic University, Dalian, 116034 P.R. China; 2 College of Microelectronics and Key Laboratory of Optoelectronics Technology, Faculty of Information Technology, Beijing University of Technology, Beijing, 100124 P.R. China; 3 Department of Electronic Engineering and State Key Laboratory of Terahertz and Millimeter Waves, City University of Hong Kong, Tat Chee Avenue, Kowloon, Hong Kong P.R. China

**Keywords:** Nanoscale materials, Nanoscale materials

## Abstract

Multivariate terbium-complexes were incorporated into polyacrylonitrile (PAN) and electrospun into flexible multifunctional nanofibers with a uniform diameter of ~200 nm. Fluorescence comparison in multi-ligand-binding nanofibers under ultraviolet (UV) radiation verifies that the differentiated β-diketone ligands with dual functions are the primary cause of the spectral fluctuation, adequately illustrating the available methods for the quantification of intermolecular reciprocities between organic ligands and central Tb^3+^ ions. Especially under 308 nm UVB-LED pumping, the total emission spectral power of supramolecular Tb-complexes/PAN nanofibers are identified to be 2.88 µW and the total emission photon number reaches to 7.94 × 10^12^ cps which are nearly six times higher than those of the binary complex ones in the visible region, respectively. By modifying the sorts of organic ligands, the luminous flux and luminous efficacy of multi-ligand Tb-complexes/PAN nanofibers are up to 1553.42 μlm and 13.72 mlm/W, respectively. Efficient photon-releasing and intense green-emission demonstrate that the polymer-capped multi-component terbium-complexes fibers have potential prospects for making designable flexible optoelectronic devices.

## Introduction

**Figure 1:**
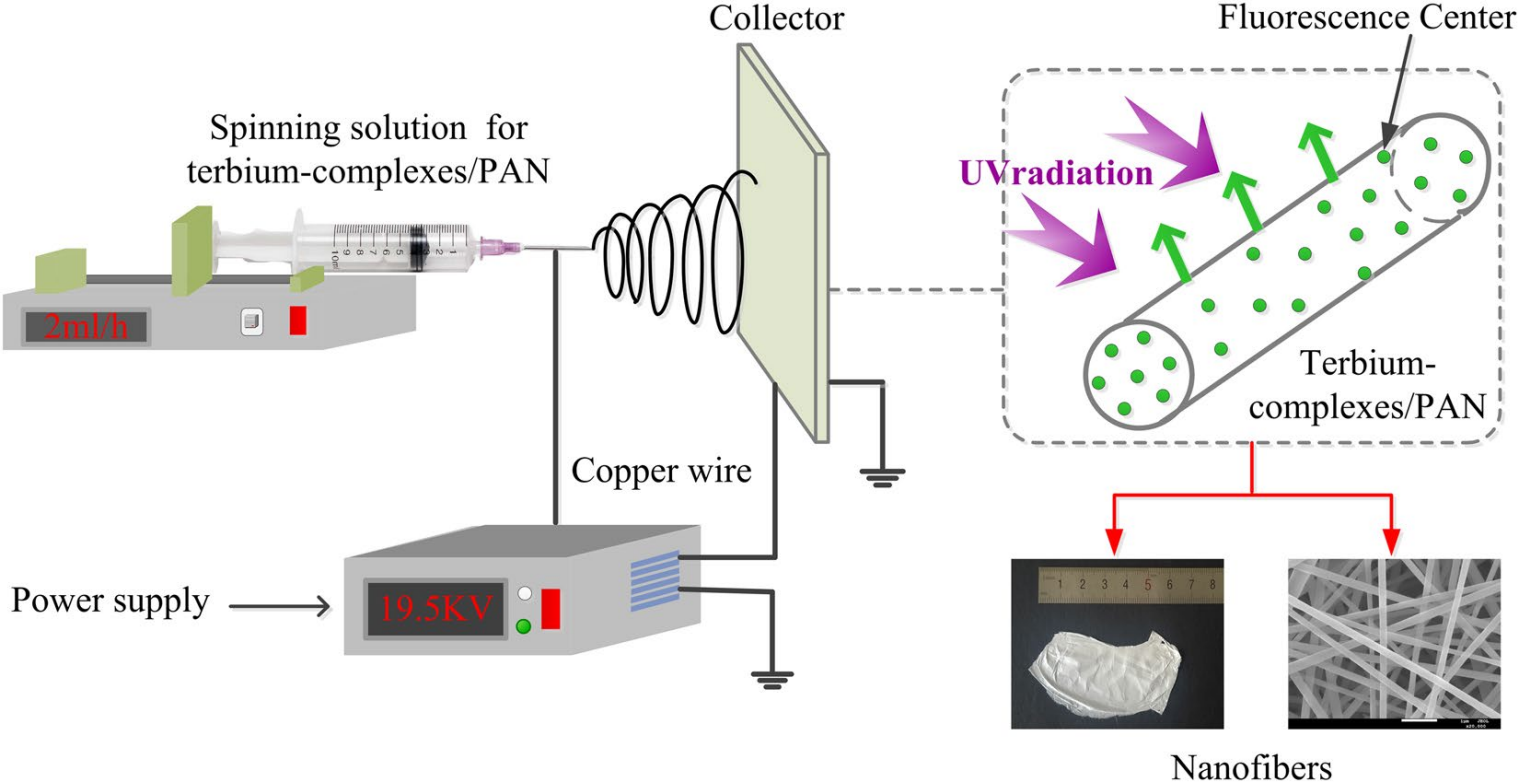
Schematic representation of the electrospun setup.

Growing interest is being devoted to exploring novel luminescent materials with assembling one-dimensional (1D) nanostructures in view of their unique properties and intriguing applications in light-emitting devices and color display^1–4^. Particularly, more remarks in challenge of 1D organic-inorganic composites lie in integrating the flexibility and processability of the organic materials into the thermal stability and chemical resistance of the inorganic materials^5–8^. So far, electrospinning is considered as a relatively straightforward method for producing 1D luminescent nanomaterials with high porosity, favorable flexibility and large surface, such as nanofibers, nanowires, nanotubes, nanorods, nanobelts and so forth^9–13^. The electrospun setup for fabricating the nanofibers is shown in Fig. 1. In comparison to other nanofibers, the ordered 1D fluorescent electrospun nanofibers with excellent photon-releasing efficiency, high surface-to-volume ratio and outstanding organic-inorganic advantages are hopeful serving in the fields of electrical, electrochemical, biomedical and environmental^14–18^.

Considering that rare earth (RE) complexes have sharp and strong emission lines under ultraviolet (UV) irradiation through effective intramolecular energy transfer from the coordinated ligands to the RE-ions, the affinity can be improved by selecting or synthesizing organic groups^19–22^. Among the trivalent lanthanide compounds with visible light emission, the investigations are mainly focused on the europium and terbium complexes which possess long-lived intense red and green luminescence with high quantum efficiency^23–26^. In addition, as a result of the absent dense energy level, the probability of non-radiative transition is greatly reduced between the triplet and ground states of ligands, thus the introduction of RE into β-diketone ligands with large absorption cross-section has attracted extensive attention^27–29^. At the same time, in order to improve inferior optical properties and poor thermal stability of pure complexes, RE-complexes are usually incorporated into polymer matrixes to embody excellent characteristics of both the complexes and the polymers and apply to various new technologies^30–34^. Although some RE-complexes doped polymer nanofibers have been investigated^35–37^, the systematic correlation on the fluctuation of photon-releasing with differentiated organic ligands in electrospun nanofibers is still worth exploring in making optoelectronic devices.

Our work has focused on the use of multifunctional ligands to capture the energy and transfer to the rare-earth-metal-ions in PAN-capped electrospun nanofiber membranes, among which the multivariate terbium-complexes nanofibers have adopted organic ligands including acetylacetone (acac), benzoic acid (BA) and 1,10-phenanthroline (Phen). Asystematic microstructural and spectroscopic characterization of poly-ligand Tb-complexes/PAN nanofibers are presented by SEM, FT-IR, fluorescence spectra, absolute spectral distributions and DSC-TGA thermodynamic investigation, which provide the evidence of excellent performance for composite fibers. Differentiated responses to short-wave radiation in these nanofibers are revealed and the energies involved in noncovalent interactions are proposed, confirming that the energy difference between the lowest triplet state energy of ligands and the resonant emissive energy of Tb^3+^ ions is chief factor which determines the luminescence performance of the swatches. Spectroscopic intensity parameters of multivariate nanofibers are derived from spectral power and photon number, as well as the luminous flux is also deducted under 308 nm UVB light emitting diode (UVB-LED) excitation, revealing the appropriate organic ligands are conducive to improving the efficiency of photon-conversion. The flexible polymeric nanofibrous membrane containing β-diketone ligands can be adopted as a developed prototype with characteristic of bending, stretching and even twisting and no negative effect on photon emission, these facilitate the development of the flexible wearable electronic devices.

## Results and Discussion

### Structure and morphology of Tb-complexes/PAN nanofibers

**Figure 2:**
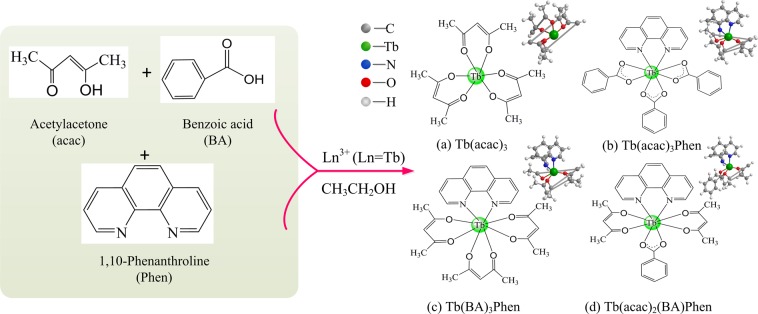
Illustration of Tb^3+^ coordination functionalized (**a**) Tb(acac)_3_, (**b**) Tb(acac)_3_Phen, (**c**) Tb(BA)_3_Phen and (**d**) Tb(acac)_2_(BA)Phen complexes, respectively.

**Figure 3:**
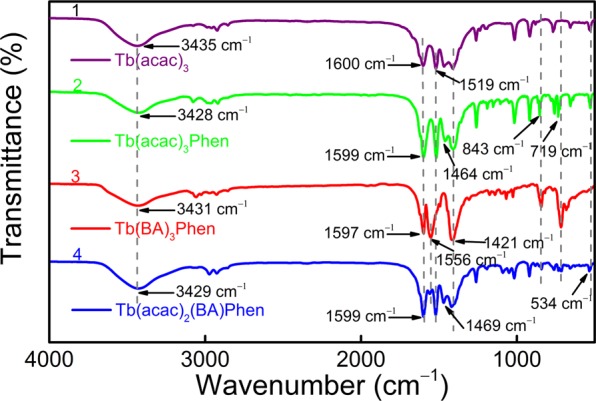
FT-IR spectra of Tb(acac)_3_ (curve 1), Tb(acac)_3_Phen (curve 2), Tb(BA)_3_Phen (curve 3) and Tb(acac)_2_(BA)Phen (curve 4) precursor powders.

In order to explore the effect of different organic ligands on the properties of composite materials, the intensive research is carried out on terbium-complex/PAN nanofibers. Figure 2 clearly determines the composition of the precursor and presents the overall molecular structures of the Tb-complexes with multi-ligand groups. Meanwhile, FT-IR spectra of Tb(acac)_3_ (curve 1), Tb(acac)_3_Phen (curve 2), Tb(BA)_3_Phen (curve 3) and Tb(acac)_2_(BA)Phen (curve 4) precursor powders have been carried out in the frequency range of 4000−400 cm^−1^ and shown in Fig. 3. For curve 1, two intense absorption bands at 1600 and 1519 cm^−1^ are associated to the enol-keto tautomers of the β-diketone corresponding to the C=O stretching vibration of keto and the C=C stretching vibration of enol, respectively, which indicates that the carboxylic acid takes off the proton to coordinate with the Tb^3+^ ions by the acid radical^38–40^. As expected, a broadband peaked at 3435 cm^−1^ may be because of the presence of external water molecule. As compared with the spectrum of curve 1, the peaks of curve 2, curve 3 and curve 4 around 1600 cm^−1^ left shift slightly corresponding to the non-characteristic absorption −N=C− stretching vibration of Phen. Meanwhile, the regions of 835–853 and 718–733 cm^−1^ in curve 2 are attributed to N–H vibration, and it is noticed that the complexation spectra at 3428 cm^−1^ appear a shift of the *δ*_(N–H)_ absorption band to a higher frequency (Δ*δ* = 7 cm^−1^) after the introduction of Phen, indicating that the chelating action from nitrogen atoms to Tb^3+^ ions has produced^41,42^. In curves 3 and 4, the peaks at 1556 and 1421 cm^−1^ are the C=O and C=C stretching vibration of β-diketone which left shift (1464 cm^−1^) due to the substitution of one or two acetylacetone by BA groups during the dissolution process^43^. Moreover, the frequency signal about 534 cm^−1^ provided valuable information about ligand-metal vibrations, which can be ascribed to the interactions of Tb–O coordination. The above spectra data fully demonstrate the formation of the multi-ligand binary (Tb(acac)_3_), ternary (Tb(acac)_3_Phen and Tb(BA)_3_Phen) and quarternary (Tb(acac)_2_(BA)Phen) complexes. β-diketone rare-earth-metal complexes not only exhibit good coordination and fine sensitization function but also possess a variety of conjugate structures^44^. In addition, the high electron cloud density of β-diketonate ligand will enable the RE-complexes to have strong photo-absorption and energy transfer ability.

**Figure 4:**
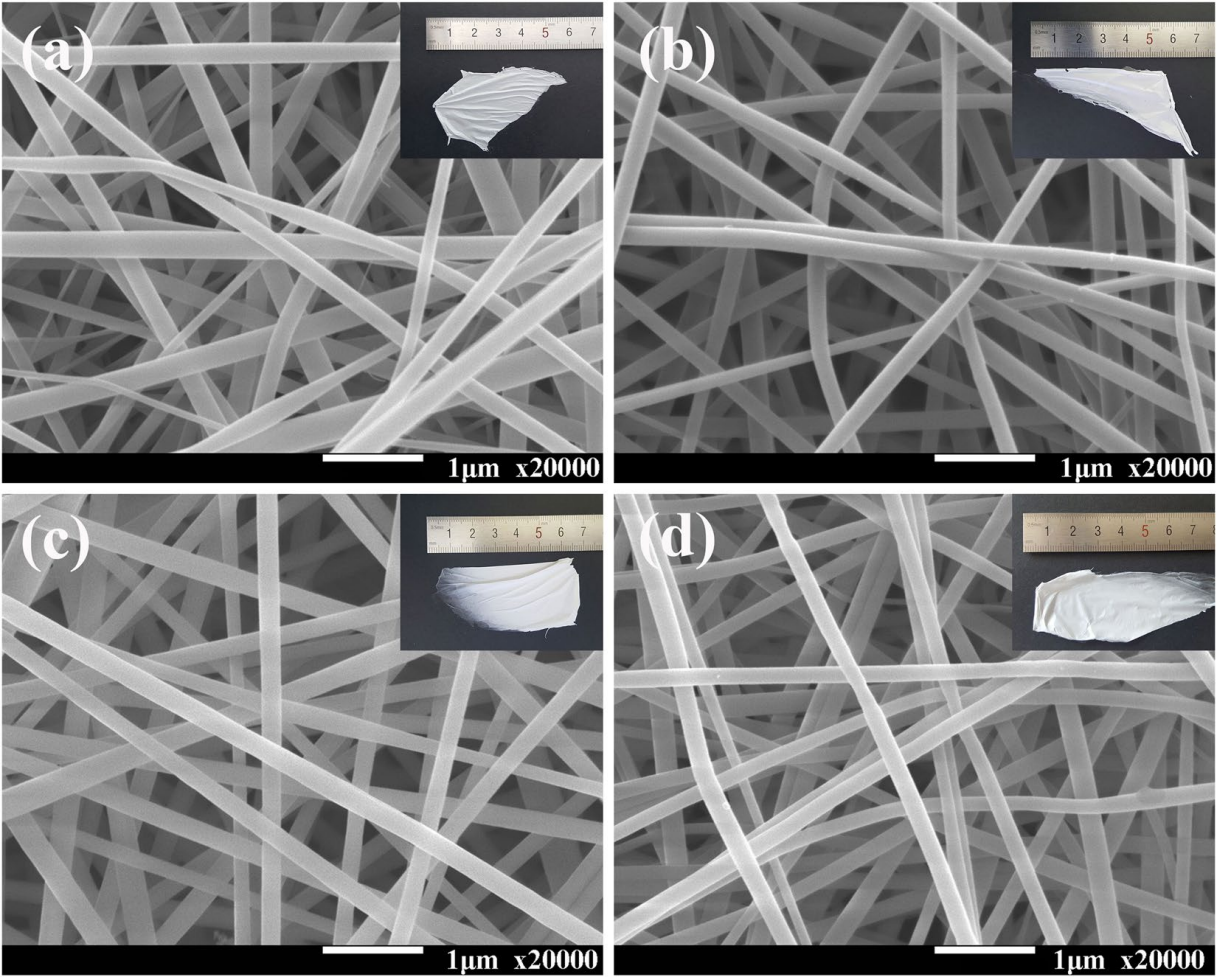
SEM micrographs of A, B, C and D electrospun nanofibers corresponding to (**a–d**) under 20000 magnification, respectively. Insets: the macro-morphology of nanofibers in natural light irradiation.

In order to further understand the micro/macro-structure of the PAN loaded Tb-complexes nanofiber membranes, the Tb(acac)_3_/PAN (A), Tb(acac)_3_Phen/PAN (B), Tb(BA)_3_Phen/PAN (C) and Tb(acac)_2_(BA)Phen/PAN (D) nanofibers with favorable morphologies are obtained as shown in Fig. 4. In addition, the insets in Fig. 4 present macro-morphology of electrospun nanofibers derived from A, B, C and D nanofibers and the fibrous membranes are white and filiform in natural light. It can be seen that all nanofibers have a uniform and smooth bead-free round morphology arranging randomly and overlapping with each other with diameters of ~200 nm.

**Figure 5:**
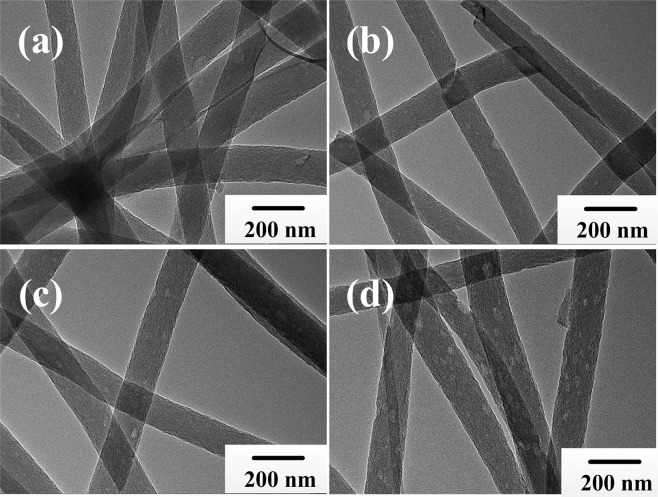
TEM images of A, B, C and D electrospun nanofibers corresponding to (**a–d**), respectively.

To further confirm and demonstrate the dispersion of precursor powders in nanofibers, the internal constructions of A, B, C and D electrospun nanofibers are clearly exhibited by TEM images in Fig. 5, indicating that the Tb-complexes are well distributed evenly in the composite systems^45,46^. Furthermore, the 1D cross-link-mesh-like superfine nanofiber also exhibits larger surface to volume ratios with a positive effect in absorbing ultraviolet (UV) and shortwave radiation, demonstrating that ordered nanoscale arrays could serve as building blocks to fabricate complex wearable optoelectronic devices and systems.

### Photoluminescence characteristics of Tb-complexes/PAN nanofibers

**Figure 6:**
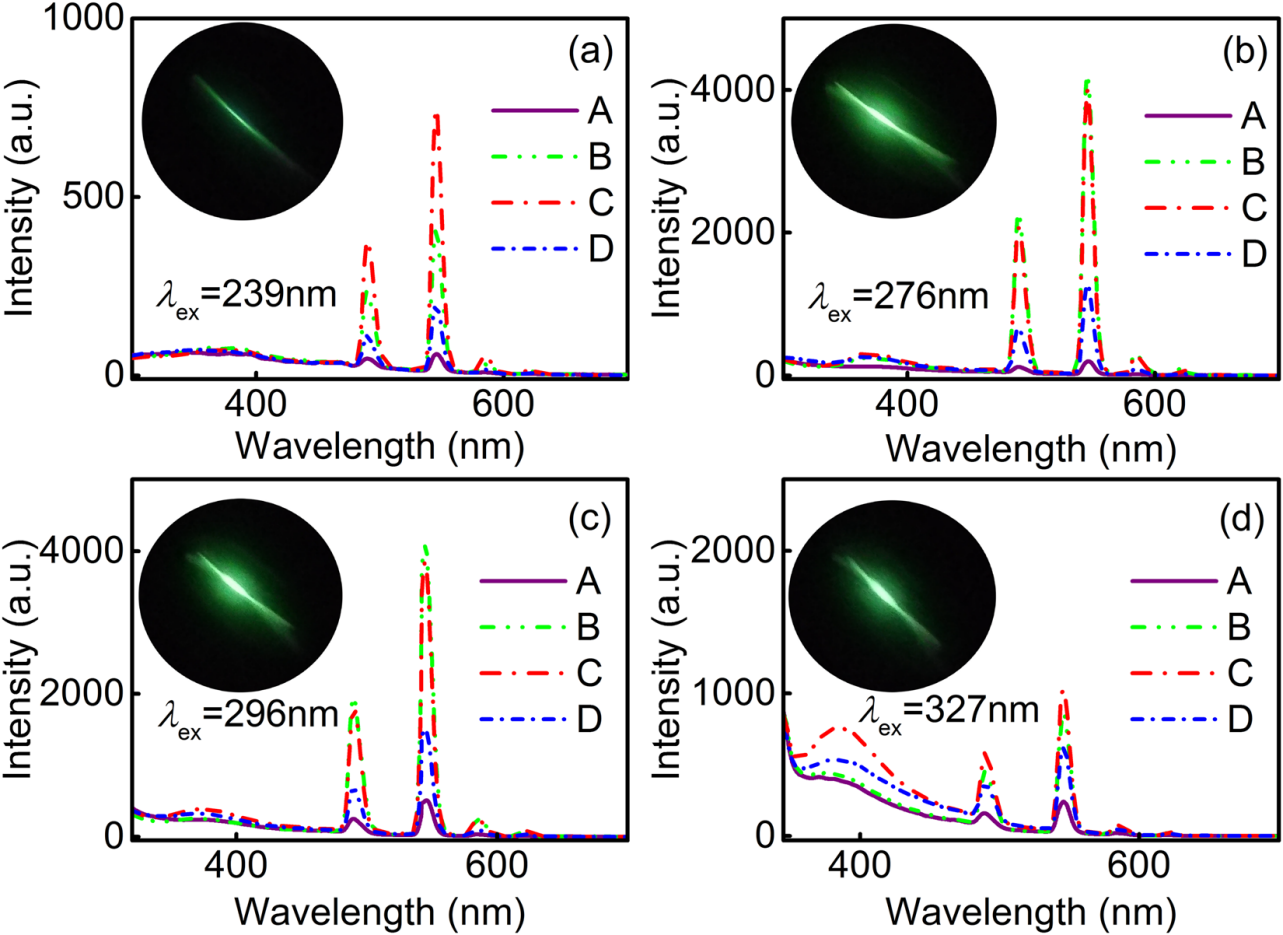
Emission spectra of A, B, C and D nanofibers excited at (**a**) 239, (**b**) 276, (**c**) 296 and (**d**) 327 nm, respectively. Insets: fluorescence photos of B nanofibers under different excitation wavelengths, respectively.

Tb^3+^ ion chelated complexes exhibit lanthanide characteristic emissions and the luminescence of the A, B, C and D nanofibers are demonstrated by the emission spectra in Fig. 6 with differential luminescence exhibited in the inserted photos, respectively. Upon different ultraviolet excitation, all spectra clearly resolve a broadband attributing to π–π^∗^ transition of ligands and emission lines of Tb^3+ 5^D_4_ → ^7^F_J_ (J = 6, 5, 4 and 3) are obtained with the hypersensitive transition ^5^D_4_ → ^7^F_4_ (545 nm) green emission as the most prominent group^47–50^. However, the emission intensity is quite different for each complex, this difference originates from the various coordination environments caused by the diverse neutral ligands.

The luminescence properties change dramatically compared with the corresponding binary complexes after the second and third ligands introducing in ternary and quaternary complexes systems. Compared of fluorescence performances between A and B nanofibers, the luminescence emission intensity is influenced greatly when Phen is introduced as a synergistic screening ligand for acac, which can explain by the difference of triplet state energies and the existence of the intermolecular reciprocity between carboxylic acids and Phen^51–53^. Bright green fluorescence is observed and the effect of Phen is confirmed upon enhanced emission, indicating the effectiveness of photon-conversion from UV to visible region. For C nanofiber, the strongest fluorescence emission is revealed under 239 and 327 nm excitations, which confirms the optimum of excitation wavelengths in the BA and Phen coexistence system. In D nanofiber, since the excited state energy of Phen is close to that of acac and lower than that of BA, the energy transfer from BA to acac and Phen leads to the decreases of the energy received and the fluorescence release of Tb^3+^ ions^54^. The variation of ligands disturbs the surrounding environment of the center Tb^3+^ ions and causes the distorted complex structure to some extent, resulting in the change of the center ion symmetry and further modified forbidden effect of f-f transition, all of which cause the discrepancy of luminescent properties in the composites.

**Figure 7:**
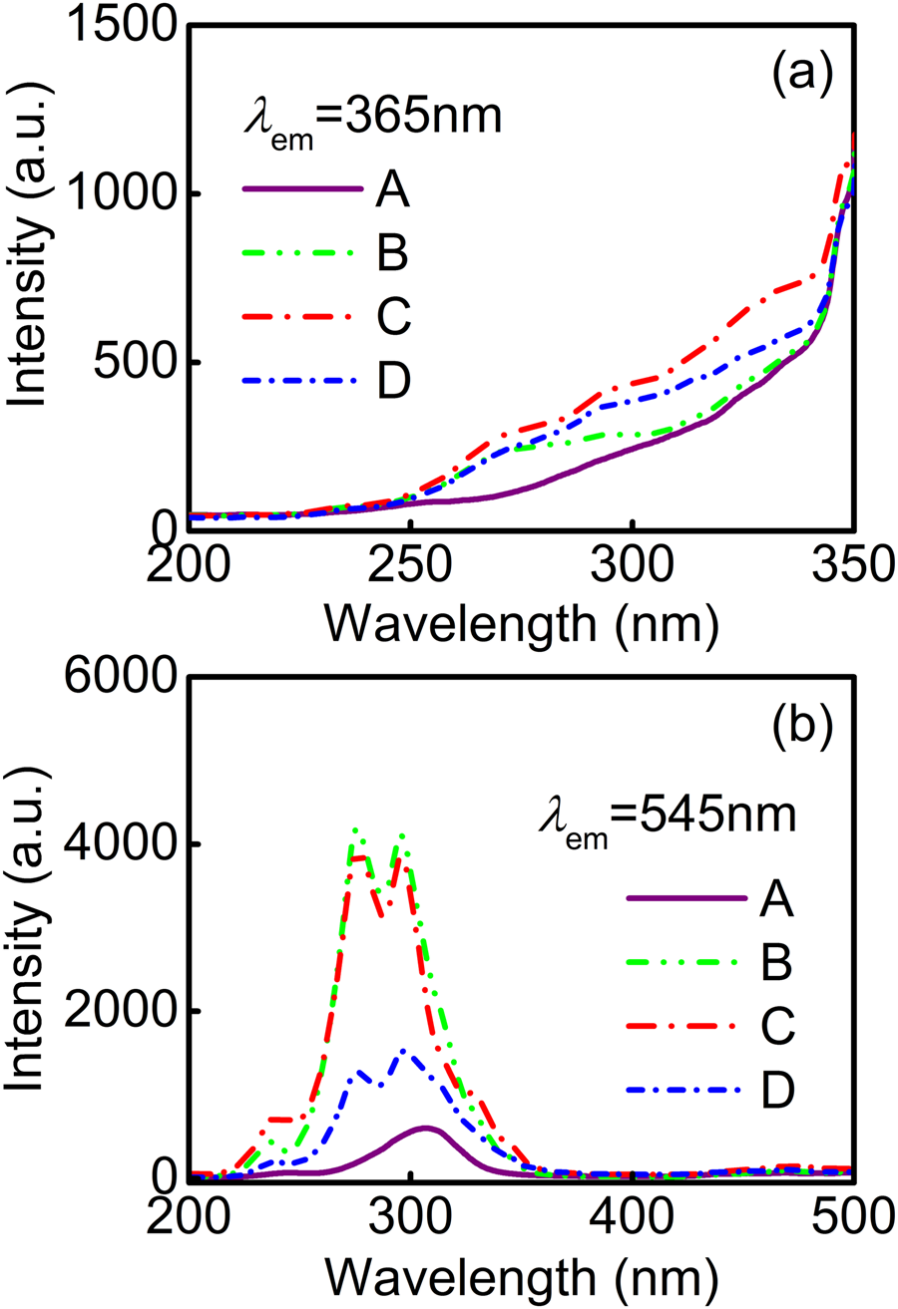
Excitation spectra of A, B, C and D nanofibers monitored at (**a)** 365 and (**b**) 545 nm, respectively.

As shown in Fig. 7, the excitation spectra of the A, B, C and D wire-shaped nanofibers are presented under the monitoring of 365 and 545 nm, respectively. With the introduction of Phen, the varied tendency of excitable intensity and the spectral shape are extremely remarkable in contrast to A nanofibers. In addition, these composite nanofibers also show obvious absorption in the near-UV wavelength region (200–400 nm), which is the characteristic absorption of the conjugated double bonds ligands originating from their efficient π → π* transition^55,56^. Except for A nanofibers, other excitation spectra monitoring the green emission of Tb^3+^ at 545 nm are dominated by a broad band centered at 276 and 296 nm, respectively, which is consistent with the emissions in all of them and indicates that the energy transfer exists between ligands and central Tb^3+^ ions. Apparently, the excitation bands of B nanofibers become wider and blue shift compared with those of the other complex nanofibers, which further demonstrates the excellent fluorescence properties in B nanofibers. However, the excitability is relatively low in case of BA, acac and Phen coexisting, which imply the radiation intensity of Tb^3+^ is subject to limitations due to the variation of energy transfer efficiency and the intermolecular interaction from the ligands to the central Tb^3+^. As the token of the increased ligand types, the absorption competition among ligands predominate, and the fluorescence intensity of the system decreases. Therefore, altering the kinds of organic ligands could change the relative intensity of Tb-complex emission to obtain strong green fluorescence under UV radiation, which further testifies that Tb^3+^ ion is suitable as the emission source of photoelectric devices.

**Figure 8:**
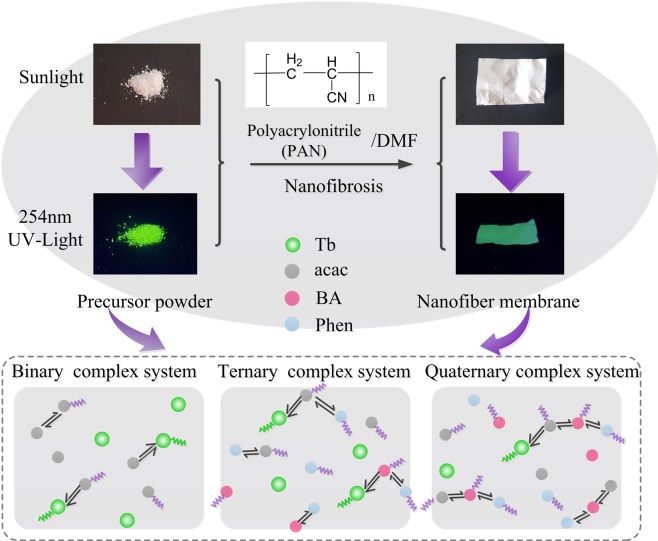
Interaction mechanism of ligand-ligand and ligand-Tb^3+^ ions in binary, ternary and quaternary complex systems.

Interaction mechanism of ligand-ligand and ligand-Tb^3+^ ions in binary, ternary and quaternary complex systems and the efficient emission photos of the nanofibers under UV exposure are portrayed in Fig. 8. Since the supersensitive transition is intensely dependent on the local asymmetry of the RE-ion environment, that is, the worse the symmetry is, the easier the supersensitive transition occurs and the stronger fluorescence obtains. Take ternary system as an example, the acac and BA ligands intensely absorb in the UV region and transfer energy to the central Tb^3+^ ions, while the Phen stabilizes the molecular structure of ternary complex system and decreases the non-radiative channels (in absence of Phen, water molecules still combine with the complex), which reveals the existence of Phen contributes to the enhancement of fluorescence intensity^57^. While in quaternary composite system, as the species of the ligands further increases, the reciprocities between ligand and ligand become more complicated and the energy loss increases, resulting in a decrease in the photon acceptability of the central Tb ions.

### Thermodynamic analysis of Tb-complexes/PAN nanofibers

**Figure 9:**
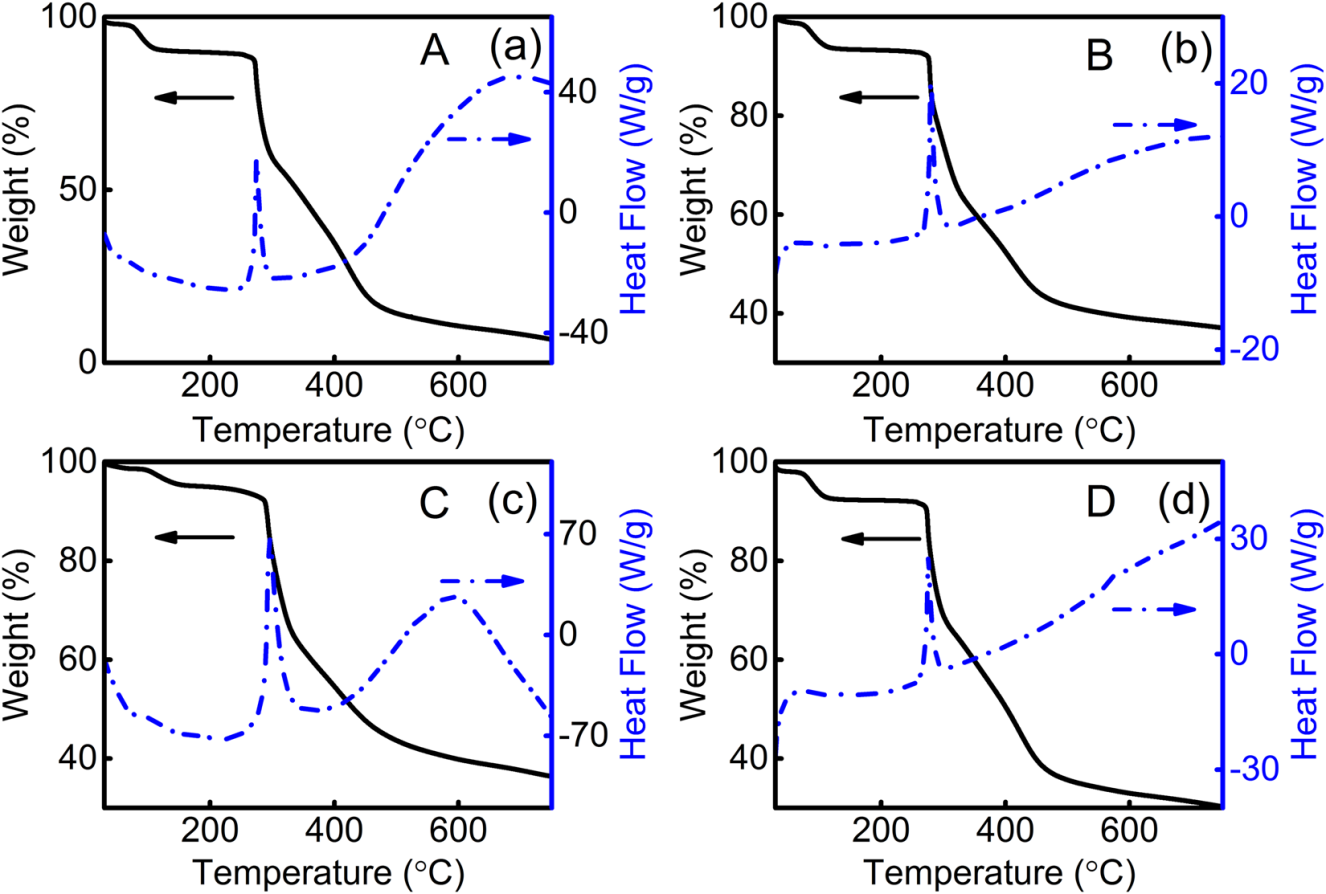
DSC-TGA curves of A, B, C and D nanofibers, respectively.

The thermal behavior of the materials involved can be further supplemented by DSC–TGA curves, and the nanofibers are put into an alumina crucible where all materials are measured in identical conditions. As can be seen from Fig. 9, the weight loss of A, B, C, and D macromolecular nanofibers mainly includes three distinct steps. Initially, the first weighing process between 35 and 150 °C with ~10% weight loss is due to the evaporation of water and the decomposition of small molecular ligands. The second weight loss gives the maximum decomposition rate at 270–290 °C with an exothermal peak appears attributing to the decomposition of PAN, which indicates that the interlinking molecular cyclization of preoxidized nanofibers is not fully accomplished^58^. Subsequently, a pre-carbonization process is further performed after 450 °C to achieve enough intermolecular cyclization. Among them, a few physical cross-links formed among the PAN molecules due to the coordination of the PAN and the Tb-complex, which plays a pivotal role in the heat resistance of the PAN-based composite nanofibers. Further, given that the operating temperature of LEDs is less than 150 °C, the macromolecular polymer-based Tb-complex nanofibers with good thermal stability are sufficient for the manufacture of LEDs^59,60^.

### Quantitative characterization and brightness analysis of Tb-complexes/PAN nanofibers

**Figure 10:**
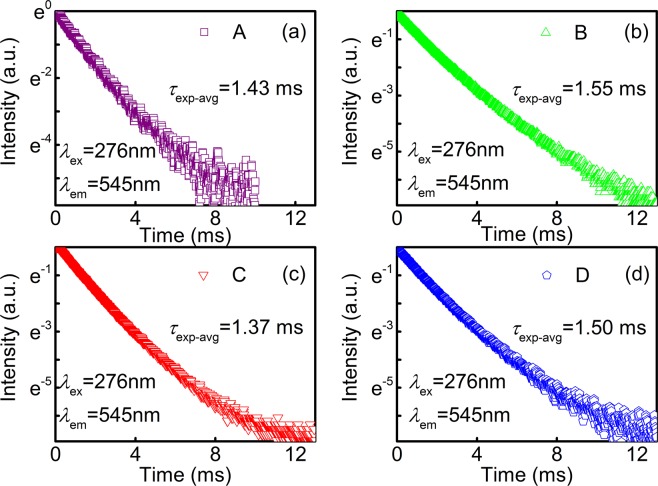
Fluorescence decay curves of the ^5^D_4_ state in A, B, C and D nanofibers monitoring at 545 nm under 276 nm excitation.

For further comparison and clarity, the fluorescence decay curves of ^5^D_4_ level in A, B, C and D nanofibers excited at 276 nm and monitored at 545 nm are presented in Fig. 10. The experimental average lifetime (*τ*_exp-avg_) of ^5^D_4_ level is derived from the fluorescence decay curves using the following equation

1 $${\tau }_{\exp -{\rm{avg}}}=\frac{{\int }_{0}^{\infty }tI(t){\rm{d}}t}{{\int }_{0}^{\infty }I(t){\rm{d}}t},$$

where *I*(*t*) is the emission intensity at time *t*. Besides, the related results are calculated and listed in Table 1. The decay curves of Tb-complexes/PAN are singly exponential, confirming that the chemical environment of Tb^3+^ is varied slightly in the complexes, from which the luminescence lifetime is determined to be 1.43, 1.55, 1.37 and 1.50 ms in A, B, C and D, respectively. The fluorescence lifetime is varied with the β-diketone ligands for Tb-complexes in organized molecular nanofibers, which can be put down to the dual functions of ligands for the Tb^3+^ ions.

**Table 1:** Total emission spectral power, total emission photon number, experimental average lifetime *τ*_exp-avg_, color coordinates, luminous flux and luminous efficacy of A, B, C and D nanofibers under the excitation of 308 nm UVB-LED with the exciting electric power of 113.2 mW.

Samples	Total emission spectral power (µW)	Total emission photon number (10^12^cps)	τ_exp-avg_ (ms)	Color coordinates (x, y)	Luminous flux (μlm)	Luminous efficacy (mlm/W)
A	0.47	1.29	1.43	(0.297, 0.625)	254.16	2.25
B	2.88	7.94	1.55	(0.322, 0.612)	1553.42	13.72
C	2.04	5.61	1.37	(0.321, 0.609)	1084.76	9.58
D	1.10	3.03	1.50	(0.332, 0.606)	594.23	5.25

To further investigate the effects of ligands on Tb^3+^ fluorescence decay in nanofibers, the fluorescence lifetime *t* of ^5^D_4_ can be expressed by the following form $t=1/({k}_{1}+{k}_{2}+{k}_{3})$, where *k*_1_ is radiation rate constant, *k*_2_ and *k*_3_ are non-radiative energy transfer and the back-donation rate constants to the ground states and to the triplet of the ligands, respectively. Among them, the resonance energy level and the rate constants *k*_1_ and *k*_2_ of Tb^3+^ are relatively stable, while the *k*_3_ could be modified with the adjustable of β-diketone ligands^61^. The longest and weakest *t* are revealed in the B and C nanofibers, which can be understood by investigating the intramolecular energy transfer process in Tb-complexes. Additionally, the *t* is also relevant to the energy transfer rate constant *k*_3_ which originates from ^5^D_4_ to the triplet of β-diketone ligands. In B nanofibers, *k*_3_ may be the smallest among those in Tb-complexes nanofibers, resulting in the longest fluorescence lifetime. Along with the introduction of BA, the fluorescence time shows a descending trend due to the triplet energy level of BA (21200 cm^−1^) is lower than those of acac (25280 cm^−1^) and Phen (22075 cm^−1^), and it is similar to the ^5^D_4_ energy level (21000 cm^−1^) of Tb^3+^ ions, causing the poor intramolecular resonance energy transfer between BA and Tb ions^62–64^. As a result, the fluorescence of Tb(acac)_3_(BA)Phen/PAN nanofiber is the weakest.

**Figure 11:**
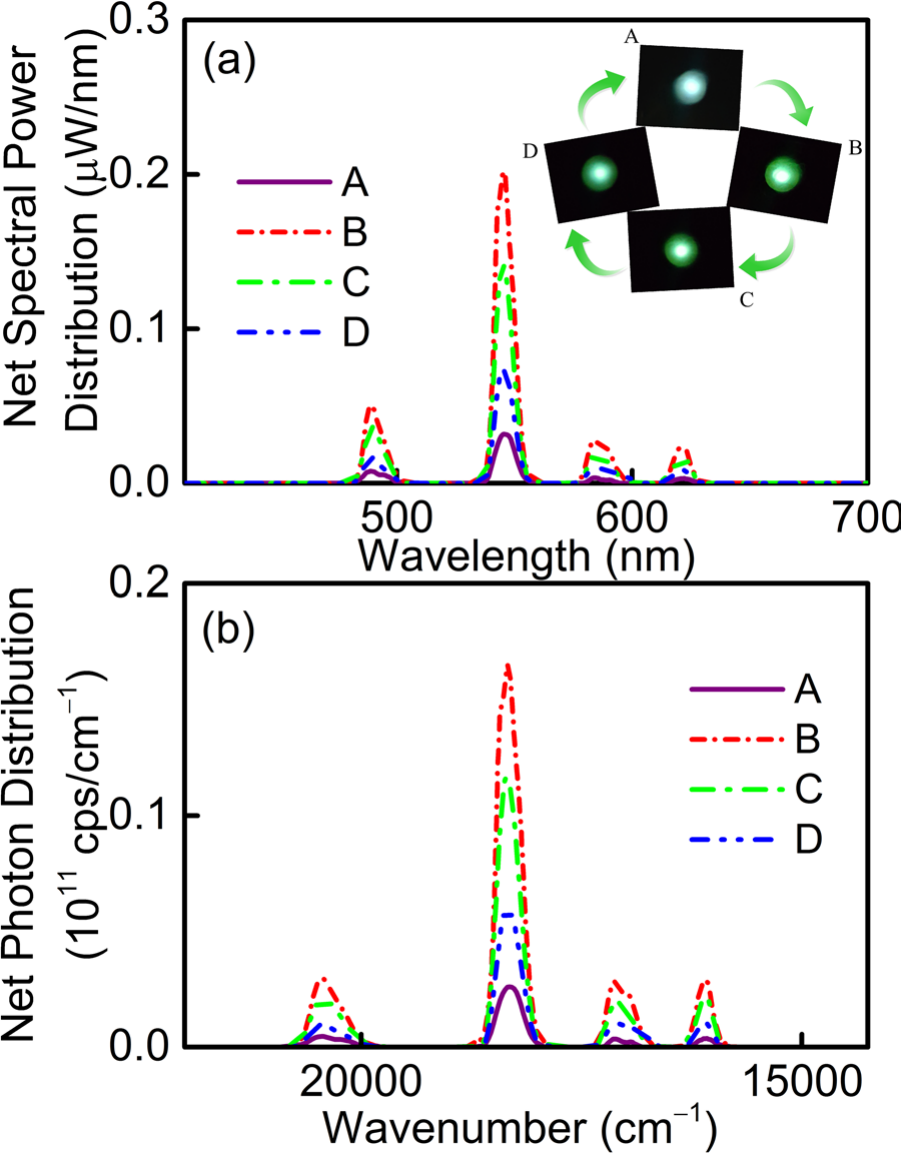
(**a**) Spectral power and (**b**) photon number distributions of A, B, C and D nanofibers under 308 nm UVB-LED excitation, respectively. Insets: fluorescence photographs of A, B, C and D nanofibers.

For a better understanding of the optical properties in these complexes, the absolute spectral parameters from those used in interiors and for which almost all data on visual performance have been accumulated. Intense green light scattering is captured in PAN-capped Tb-complexes and spectral power distributions of A, B, C and D nanofibers are recorded using an integrating sphere with a 308 nm UVB-LED as the pump source, which consists of a weak broadband and four major luminescent peaks centering at 480, 545, 584 and 621 nm originate from the ^5^D_4_→^7^F_J_ (J = 6, 5, 4 and 3) transitions with the strongest emission at 545 nm as shown in Fig. 11(a), respectively^65,66^. In the meantime, the spectra are reshaped gradually and the emitting light of composite nanofibers is tuned by modifying the class of β-diketone ligands in Tb-complexes. Besides, the total emission powers of A, B, C and D nanofibers are identified to be 0.47, 2.88, 2.04 and 1.10 µW, respectively. In view of the increasing interest in systems capable of generating photon fields containing a preset number of photons, it chiefly provides basic information and related applications in the field of optics. Furthermore, the photon distribution can be given by the following formula

2 $$N(v)=\frac{{\lambda }^{3}}{hc}P(\lambda ),$$

where *λ*, *ν*, *h* and *c* represent wavelength, wavenumber, Planck’s constant and vacuum light velocity, respectively. The total emission photon numbers are derived to be 1.29 × 10^12^, 7.94 × 10^12^, 5.61 × 10^12^ and 3.03 × 10^12^ cps for A, B, C and D nanofibers, respectively, and the photon distribution curves are presented in Fig. 11(b). Besides, it is clear that the photon number distribution intensity reaches the maximum for B which is nearly six times higher than those of the binary complex ones and further confirms the well-matching resonance energy level between ligands and Tb^3+^ ions in B nanofibers.

**Figure 12:**
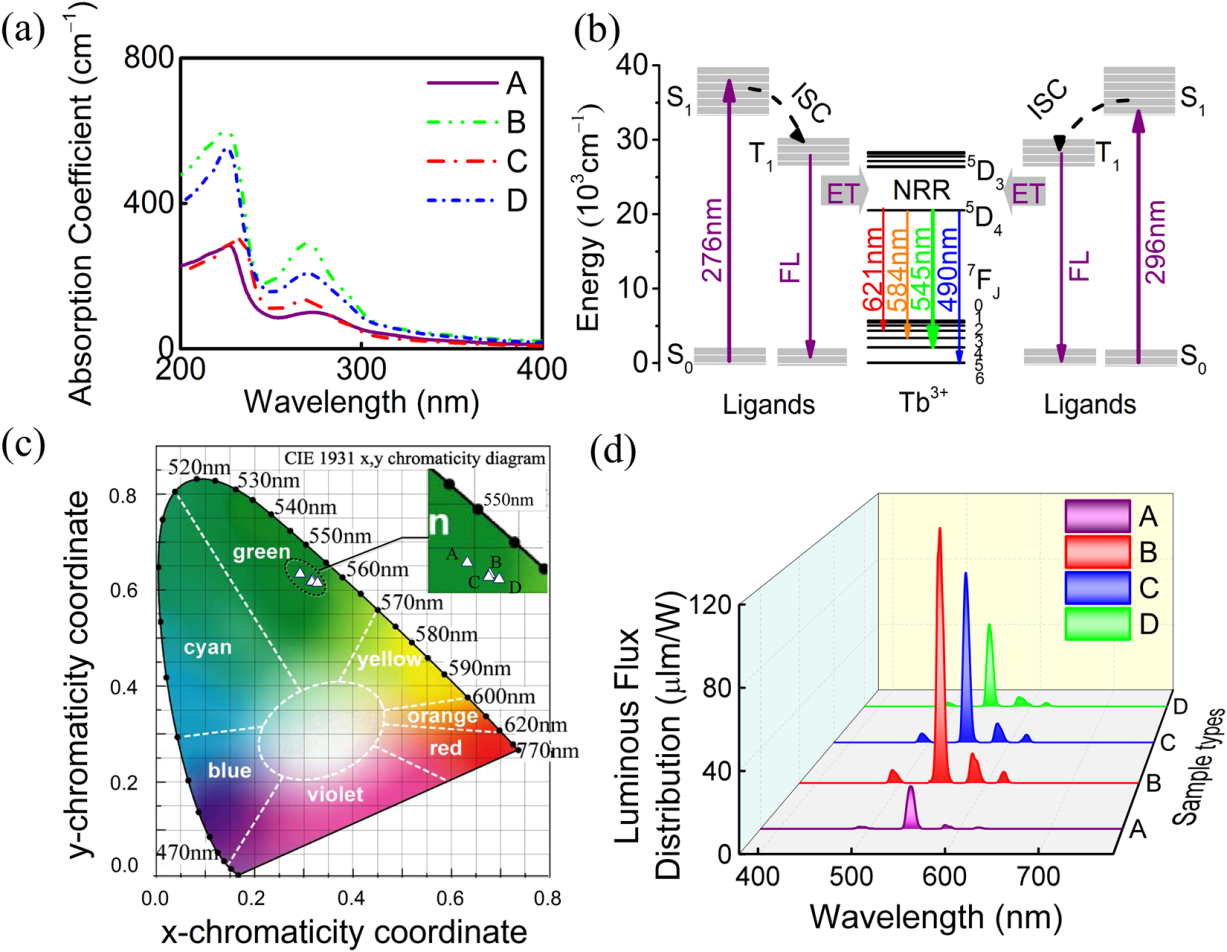
(**a**) Absorption spectra of A, B, C and D nanofibers, respectively. (**b**) Energy transfer mechanism of Tb-complex nanofibers. (**c**) CIE chromaticity diagram of nanofibers under the 308 nm UVB-LED excitation. (**d**) Luminous flux distributions of A, B, C and D nanofibers with the excitation electric power of 113.2 mW. (ET = Energy transfer, ISC = Inter-system crossing, NRR = Non-radiative relaxation).

Besides, UV absorption of RE-complexes is mainly determined by ligands. When the RE-ions bond with ligands, the light absorptivity increases due to the enhance of electron delocalization and oxygen atoms conjugation and the decrease of the system energy. Here, the UV-Vis absorption spectra of A, B, C and D nanofibers are presented in Fig. 12(a). It is evident that the ligand group has an obvious influence on its absorption capacity and the broadband at 200–350 nm indicates the strong absorbability of ligands, demonstrating the PAN is an appropriate substrate for photon conversion materials doped with RE-complexes. It is observed that one absorption peak at 225–234 nm is surveyed which is related to the n → σ^*^ transition of β-diketonate ligand, and another absorption band at 274 nm is attributed to the π → π^*^ transition of the non-bonded electrons involving the oxygen atoms. The absorption peak for B, C and D nanofibers at 270 nm is primarily ascribed to the superimposition of n → π^*^ transition of C=N in Phen and the π → π^*^ electron transition of the benzene ring^67^. Moreover, an obvious blue shift is observed in the spectra after the addition of Phen owing to a large resonance effect of the substituent between the carboxylate groups and N atoms and an increase of electron conjugation in lanthanide metal centers, which increases the π^*^ level to a higher extent than the n rank^68^. These results show a maximum photo-absorption capacity of polymer-Tb composite nanofiber and confirm the existence of intramolecular interaction and sensitization effect, further demonstrating that the fibrous membranes could be well excited at the short-wavelength UV region.

Energy transfer efficiency is primarily determined by the matching degree between the triplet state of the ligands and the excited state of RE-ions. Figure 12(b) is a generally accepted energy diagram showing how the energy is absorbed, transferred and eventually released into fluorescence (FL). Absorption efficiency, ligand-to-rare-earth energy transfer and rare-earth photon conversion determine the luminescence properties of the material, thus the effect of organic ligands is considerable in this process^64,69,70^. Therefore, an efficient energy transfer (ET) mechanism among the ligands is also discussed in this part. Taking the dominance of ligand radiation under 276 and 296 nm excitations as examples, the ligand absorbs energy from the ground state (S_0_) to the excited singlet state (S_1_), then inter-system crossing (ISC) to the excited triplet state (T_1_) and then releases FL. In addition, the neutral ligands may compete with the central ligands to absorb energy in the presence of multiple neutral ligands. Therefore, compared with binary and ternary complexes, the reduced luminescence intensity in the quaternary complex nanofibers is due to the disturbed energy absorption process and the lost energy between different ligands. Afterward the ET occurs from the ligands to the resonantly excited ^5^D_3_ level of Tb^3+^ ions, and the excited ions decay to the energetically ^5^D_4_ states through multi-phonon relaxation and then the characteristic fluorescence is released via the non-radiative relaxation (NRR)^71,72^. When it comes to color displays, the CIE-1931 chromaticity coordinates of the green color shift from (0.297, 0.625) to (0.322, 0.612), (0.321, 0.609) and (0.332, 0.606) with the amelioration of ligands in A, B, C and D nanofibers with the introduction of more components and the CIE diagram is represented in Fig. 12(c), respectively, which represents good color quality and further provides relatively reliable basis for predicting luminescence properties of the polymeric nanofibers containing β-diketone ligands in optoelectronics fields.

Luminous efficacy provides a relation between daylight and solar radiation, which reflects valuable insight into the spectral quality of incident radiation, in other words, it is helpful information for the research of optoelectronic materials^73^. Under the 308 nm UVB-LED excitation, the total luminous flux Φ_v_ of A, B, C and D nanofibers can be defined by the following expression

3 $${\Phi }_{{\rm{V}}}={K}_{{\rm{m}}}{\int }_{380}^{780}V(\lambda )P(\lambda ){\rm{d}}\lambda ,$$

where *V*(*λ*) and *K*_m_ are the relative eye sensitivity and the maximum luminous efficacy at 555 nm (683 lm/W), respectively. All of the emission spectra offer very high luminous efficacy as illustrated in Fig. 12(d), the relevant total luminous fluxes Φ_v_ and the luminous efficacy are exposed in Table 1.

Under the 308 nm UVB-LED excitation with the exciting electric power of 113.2 mW, the total luminous flux and the luminous efficacy of B nanofibers reach to the maximum 1553.42 μlm and 13.72 mlm/W, respectively, which surpass those of other nanofibers with an accordance in fluorescence spectra. Meanwhile, the brightness parameter of B is higher than that of A, C and D nanofibers, which is mainly owing to the better interactivity between ligands and RE ions, further indicating that the B nanofibers possess stronger sensitivity of the intramolecular energy transfer between the triplet state energy of ligands and the resonant emissive energy of central Tb^3+^ ions. Therefore, the polymer-based Tb-complex nanofibers provide an orientation for the systematic design and development of advanced functional materials by ligand optimization and electrospun fibrosis.

## Conclusions

Superfine and uniform multivariate β-diketone terbium-complexes integrated polyacrylonitrile (PAN) nanofibers are prepared via electrospun with a diameter of ~200 nm and bright green emission is obtained under ultraviolet excitation. Fluctuated photon releasing with multi-component β-diketone ligands compounds is attributed to the suitable energy matching between the resonance energy levels of the central Tb^3+^ ions and the lowest triplet states of the ligands. The total emission powers of Tb(acac)_3_, Tb(acac)_3_Phen, Tb(BA)_3_Phen and Tb(acac)_2_(BA)Phen doped PAN nanofibers are identified to be 0.47, 2.88, 2.04 and 1.10 µW, and the total emission photon numbers are up to 1.29 × 10^12^, 7.94 × 10^12^, 5.61 × 10^12^ and 3.03 × 10^12^ cps under the excitation of 308 nm UVB-LED, respectively. Furthermore, the luminous fluxes and the fluorescence lifetimes are recorded under short-wavelength radiation, revealing the intramolecular interaction and sensitization of ligands have great influence on the photon-conversion of Tb-complexes/PAN nanofibers. Efficient photon releasing and positive emission behavior for polymer-based Tb-complex composites provide a positive role for promoting the development of the advanced flexible photoelectronic devices.

## Methods

### Fabrication of multivariate terbium-complexes nanofibers

Precursor powders of Terbium-complexes Tb(acac)_3_, Tb(acac)_3_Phen, Tb(BA)_3_Phen and Tb(acac)_2_(BA)Phen were synthesized with acetylacetone (acac), benzoic acid (BA) and 1,10-phenanthroline monohydrate (Phen) as ligands, respectively. For instance, according to Tb(acac)_2_(BA)Phen chemical formula, TbCl_3_·6H_2_O, acac, BA and Phen were dissolved in 40 mL 99.7% ethanol in a molar ratio of 1:2:1:1, and then sodium hydroxide (NaOH) was added to adjust the PH to 6–7. Subsequently, polyacrylonitrile (PAN) were dissolved in N, N-Dimethylformamide (DMF) solutions with Tb(acac)_3_, Tb(acac)_3_Phen, Tb(BA)_3_Phen and Tb(acac)_2_(BA)Phen precursors concentration occupied 1 wt% of PAN weight and then labeled as A, B, C and D, respectively. All the Tb-complex/PAN electrospinning solutions were magnetically stirred until uniform and transparent at room temperature. During the electrospinning process, a 19.5 kV voltage was provided by a variable high DC voltage power and the 20 cm distance was setup between the nozzle and the collector.

### Measurements and characterization

The transmission spectra of Tb-complex precursors were investigated by a Perkin-Elmer FTIR/NIR Spectrometer (FT-IR), which reflected the functional groups in complexes for wavenumbers of 4000–400 cm^−1^ using KBr pellets. Field-emission scanning electron microscope (SEM instrument, JEOL JSM-7800F) was employed to observe the surface morphologies of the terbium-complexes doped PAN nanofibrous membranes at 5 kV accelerating voltage. Transmission electron microscopy (TEM, JEM-2100F) was studied to confirm dispersion of composite precursors in nanofibers. Photoluminescence spectra and fluorescence decay curves were recorded with a Hitachi F-7000 fluorescence spectrophotometer, using a R928 photomultiplier (PMT) tube as the detector and a commercial Xe-lamp as the excitation source. Differential scanning calorimetry (DSC) and the thermogravimetric analysis (TGA) were carried out to study the thermal performance by an American TA company SDT 600 under N_2_ atmosphere with a heating rate of 20 °C/min. The absorption spectra were conducted by a Perkin-Elmer UV/VIS/NIR Lambda 950 double-beam spectrophotometer. Absolute spectral parameters of the samples were obtained in an integrating sphere (Labsphere) with an internal diameter of 25 cm equipped a QEP01780 standard CCD detector (Ocean Optics) with a core wire of 600 μm. Under 308 nm UVB-LED excitation, the excited current was fixed at 20 mA and the referenced voltage was 5.66 V. A standard halogen lamp (Labsphere, SCL-050) was used to calibrate the measurement system and the nanofibers were placed on the top of the LED.

## Data Availability

All data regarding the work presented here is available upon reasonable request to the corresponding author.

## References

[1] Cui TT (2018). Facile access to wearable device via microfluidic spinning of robust and aligned fluorescent microfibers. ACS Appl. Mater. Inter..

[2] Zhang J (2018). Flexible inorganic core-shell nanofibers endowed with tunable multicolor upconversion fluorescence for simultaneous monitoring dual drug delivery. Chem. Eng. J..

[3] Schoolaert E, Hoogenboom R, Clerck KD (2017). Colorimetric nanofibers as optical sensors. Adv. Funct. Mater..

[4] Xue JJ, Xie JW, Liu WY, Xia YN (2017). Electrospun nanofibers: new concepts, materials, and applications. Accounts Chem. Res..

[5] Wang F, Wu YD, Huang YD, Liu L (2018). Strong, transparent and flexible aramid nanofiber/POSS hybrid organic/inorganic nanocomposite membranes. Compos. Sci. Technol..

[6] Ye D (2018). Large-Scale Direct-Writing of Aligned Nanofibers for Flexible Electronics. Small.

[7] Shi JH (2019). Polyvinylpyrrolidone Nanofibers Encapsulating an Anhydrous Preparation of Fluorescent SiO_2_–Tb^3+^ Nanoparticles. Nanomaterials.

[8] Awada H (2019). Controlled Anchoring of Iron Oxide Nanoparticles on Polymeric Nanofibers: Easy Access to Core@ Shell Organic-Inorganic Nanocomposites for Magneto-Scaffolds. ACS Appl. Mater. Inter..

[9] Arasu V, Hwang S, Zhang B, Byun D, Park SH (2019). 1D Fibers and 2D Patterns Made of Quantum Dot-Embedded DNA via Electrospinning and Electrohydrodynamic Jet Printing. Adv. Mater. Technol..

[10] Lei TP, Xu ZJ, Cai XM, Sun DH (2018). New Insight into Gap Electrospinning: Toward Meter-long Aligned Nanofibers. Langmuir.

[11] Wang CY (2019). Fabrication of electrospun polymer nanofibers with diverse morphologies. Molecules.

[12] Yoon J, Yang HS, Lee BS, Yu WR (2018). Recent progress in coaxial electrospinning: new parameters, various structures, and wide applications. Adv. Mater..

[13] Zhou XQ (2016). Self-Assembly of Hierarchical Chiral Nanostructures Based on Metal-Benzimidazole Interactions: Chiral Nanofibers, Nanotubes, and Microtubular Flowers. Small.

[14] Aliheidari N, Aliahmad N, Agarwal M, Dalir H (2019). Electrospun Nanofibers for Label-Free Sensor Applications. Sensors.

[15] Yen HJ, Chang CW, Wong HQ, Liou GS (2018). Cyanotriphenylamine-based polyimidothioethers as multifunctional materials for ambipolar electrochromic and electrofluorochromic devices, and fluorescent electrospun fibers. Polym. Chem-UK.

[16] Choi SJ (2017). Electrospun nanostructures for high performance chemiresistive and optical sensors. Macromol. Mater. Eng..

[17] Yang G (2018). From nano to micro to macro: Electrospun hierarchically structured polymeric fibers for biomedical applications. Prog. Polym. Sci..

[18] Pascariu P (2019). Novel rare earth (RE-La, Er, Sm) metal doped ZnO photocatalysts for degradation of Congo-Red dye: Synthesis, characterization and kinetic studies. J. Environ. Manage..

[19] Li Y, Zhang CY, Yu DG, Wang X (2016). Tailoring spatial distribution of Eu (TTA)_3_phen within electrospun polyacrylonitrile nanofibers for high fluorescence efficiency. RSC Adv..

[20] Manzani D (2018). Luminescent silicone materials containing Eu^3+^-complexes for photonic applications. J. Mater. Chem. C.

[21] Wang MS, Guo GC (2016). Inorganic-organic hybrid white light phosphors. Chem. Commun..

[22] Khuyen H. T. *et al*. Luminescent and magnetic properties of multifunctional europium (III) complex based nanocomposite. *J. Rare Earth*. (2019).

[23] Hasan N, Iftikhar K (2019). Syntheses, crystal structure and photophysical properties of [Sm(dbm)_3_(impy)] and [Tb(dbm)_3_(impy)] and their hybrid films. New J. Chem..

[24] Zhao JT, Huang LH, Zhao SL, Xu SQ (2019). Enhanced luminescence in Tb^3+^−doped germanate glass ceramic scintillators containing CaF_2_ nanocrystals. J. Am. Ceram. Soc..

[25] Pan GC (2017). Doping lanthanide into perovskite nanocrystals: highly improved and expanded optical properties. Nano Lett..

[26] Zouzou A (2018). Synthesis, structure and magnetic investigations of dinuclear lanthanide complexes based on 2-ethoxycinnamate. Dalton T..

[27] Chu XY, Wang WM, Nie YY, Cui JZ, Gao HL (2018). Regulating the luminescent and magnetic properties of rare-earth complexes with β-diketonate coligands. New J. Chem..

[28] Dandekar MP, Itankar SG, Kondawar SB, Nandanwar DV, Koinkar P (2018). Photoluminescent electrospun europium complex Eu(TTA)_3_phen embedded polymer blends nanofibers. Opt. Mater..

[29] Zhu XC (2018). Versatile reactivities of rare-earth metal dialkyl complexes supported by a neutral pyrrolyl-functionalized β-diketiminato ligand. Dalton T..

[30] Enizi AMA, Zagho MM, Elzatahry AA (2018). Polymer-based electrospun nanofibers for biomedical applications. Nanomaterials.

[31] Upadhyay A, Karpagam S (2019). Movement of new direction from conjugated polymer to semiconductor composite polymer nanofiber. Rev. Chem. Eng..

[32] Kausar A (2019). Polyacrylonitrile-based nanocomposite fibers: A review of current developments. J. Plast. Film Sheet..

[33] Fan LB (2018). Conjugate electrospinning-fabricated nanofiber yarns simultaneously endowed with bifunctionality of magnetism and enhanced fluorescence. J. Mater. Sci..

[34] Miluski P, Kochanowicz M, Zmojda J, Ragin T, Dorosz D (2019). Spectroscopic investigation of Tb(tmhd)_3_-Eu(tmhd)_3_ co-doped poly (methyl methacrylate) fibre. Opt. Mater..

[35] Liu YW (2016). Flexible hollow nanofibers: Novel one-pot electrospinning construction, structure and tunable luminescence-electricity-magnetism trifunctionality. Chem. Eng. J..

[36] Wen SP, Zhang R, Hu S, Zhang LQ, Liu L (2015). Improved fluorescence properties of core-sheath electrospun nanofibers sensitized by silver nanoparticles. Opt. Mater..

[37] He X (2017). Efficient energy transfer in terbium complexes/porous boron nitride hybrid luminescent materials. J. Phys. Chem. C.

[38] Lv F. Z., Zhang Y. H., Chen X. & Ma Y. Composition and Fluorescence of Gadolinium (III) Acetylacetonate Derivatives by Solvothermal Method. *International Journal of Optics and Photonic Engineering*. **2** (2017).

[39] Marandi F (2018). Treatment of cadmium (II) and zinc (II) with N_2_-donor linkages in presence of β-diketone ligand; supported by structural, spectral, theoretical and docking studies. Inorg. Chim. Acta.

[40] Wang D (2015). Luminescent polymethacrylate composite nanofibers containing a benzoic acid rare earth complex: morphology and luminescence properties. Dyes Pigments.

[41] Kumar B, Kaur G, Rai SB (2017). Acetylsalicylic acid sensitized lasing luminescence of terbium complex in PVA: a case of energy avalanche via 1, 10-Phenanthroline. J. Photoch. Photobiolo. A.

[42] Matsushita AFY, Pais AACC, Valente AJM (2019). Energy transfer and multicolour tunable emission of Eu, Tb (PSA) Phen composites. Colloid. Surface. A.

[43] He X (2018). Porous boron nitride/rare earth complex hybrids with multicolor tunable photoluminescence. J. Alloys Compd..

[44] Liu DT (2015). Highly Isoselective Coordination Polymerization of ortho-Methoxystyrene with β-Diketiminato Rare-Earth-Metal Precursors. Angew. Chem. Int. Edit..

[45] Daniela DC (2013). Near-field electrospinning of light-emitting conjugated polymer nanofibers. Nanoscale.

[46] Kuo CC, Lin CH, Chen WC (2007). Morphology and photophysical properties of light-emitting electrospun nanofibers prepared from poly (fluorene) derivative/PMMA blends. Macromolecules.

[47] Marques LF (2016). Energy transfer process in highly photoluminescent binuclear hydrocinnamate of europium, terbium and gadolinium containing 1, 10-phenanthroline as ancillary ligand. Inorg. Chim. Acta.

[48] Yan GW, Zhang Y, Di WH, Qin WP (2018). Synthesis of luminescent CePO_4_: Tb/Au composite for glucose detection. Dyes Pigments.

[49] Xiao W, Liu XF, Zhang JH, Qiu JR (2019). Realizing Visible Light Excitation of Tb^3+^ via Highly Efficient Energy Transfer from Ce^3+^ for LED-Based Applications. Adv. Opt. Mater..

[50] Wang Z (2016). Fabrication of superhydrophobic and luminescent rare earth/polymer complex films. Sci. Rep-UK.

[51] Jiao JQ (2018). NaYbF_4_: Tb/Eu modified with organic antenna for improving performance of polymer solar cells. Electrochim. Acta.

[52] Varaksina EA (2018). Influence of fluorinated chain length on luminescent properties of Eu^3+^ β-diketonate complexes. J. Lumin..

[53] Bala M (2018). Synthesis, photoluminescence behavior of green light emitting Tb (III) complexes and mechanistic investigation of energy transfer process. J. Fluoresce..

[54] Kim D (2015). Luminescent properties of rare earth fully activated apatites, LiRE_9_(SiO_4_)_6_O_2_ (RE= Ce, Eu, and Tb): site selective crystal field effect. Inorg. Chem..

[55] Gao BJ, Zhang DD, Li YB (2018). Synthesis and photoluminescence properties of novel Schiff base type polymer-rare earth complexes containing furfural-based bidentate Schiff base ligands. Opt. Mater..

[56] Andiappan K (2018). *In vitro* cytotoxicity activity of novel Schiff base ligand-lanthanide complexes. Sci. Rep-UK.

[57] Shahi PK, Singh AK, Singh SK, Rai SB, Ullrich B (2015). Revelation of the technological versatility of the Eu (TTA)_3_Phen complex by demonstrating energy harvesting, ultraviolet light detection, temperature sensing, and laser applications. ACS Appl. Mater. Inter..

[58] Gao HL, Huang SX, Zhou XP, Liu Z, Cui JZ (2018). Magnetic properties and structure of tetranuclear lanthanide complexes based on 8-hydroxylquinoline Schiff base derivative and β-diketone coligand. Dalton T..

[59] Sun NQ (2015). Synthesis, characteristics and luminescent properties of a new Tb (III) ternary complex applied in near UV-based LED. Opt. Mater..

[60] Zhao FL (2015). Luminescent polymethacrylate composite nanofibers containing a benzoic acid rare earth complex: morphology and luminescence properties. J. Alloys Compd..

[61] Brown TD, Shepherd TM (1973). Factors affecting the quantum efficiencies of fluorescent terbium (III) chelates in the solid state. Dalton T..

[62] Zhang RJ, Yang KZ, Yu AC, Zhao XS (2000). Fluorescence lifetime and energy transfer of rare earth β-diketone complexes in organized molecular films. Thin Solid Films.

[63] Sager WF, Filipescu N, Serafin FA (1965). Substituent effects on intramolecular energy transfer. I. Absorption and phosphorescence spectra of rare earth β-diketone chelates. J Phys. Chem..

[64] Yan B, Zhang HJ, Wang SB, Ni JZ (1998). Intramolecular energy transfer mechanism between ligands in ternary rare earth complexes with aromatic carboxylic acids and 1, 10-phenanthroline. J. Photoch. Photobiolo. A.

[65] Chanu TTT, Singh NR (2018). Investigation on optical band gap, photoluminescence properties and concentration quenching mechanism of Pb_1−x_Tb^3+^_x_WO_4_ green-emitting phosphors. Spectrochim. Acta A.

[66] Zhao ZW (2019). Adjustable multicolor emission from the combination of up-conversion in Tm^3+^/Tb^3+^/Yb^3+^ tri-doped Na_5_Lu_9_F_32_ single crystals. Opt. Mater..

[67] Wang Q (2018). Structure and photoluminescence property of Eu, Tb, Zn-containing macromolecular complex for white light emission. Opt. Laser Technol..

[68] Gao XC (1999). Photoluminescence and electroluminescence of a series of terbium complexes. Synthetic Met..

[69] Bai JY, Gu HQ, Hou YJ, Wang SH (2018). Luminescence properties and molecular mechanics calculation of bis-β-diketonate Eu^3+^ complex/polymer hybrid fibers. Opt. Mater..

[70] Xin H (2004). The effect of different neutral ligands on photoluminescence and electroluminescence properties of ternary terbium complexes. J. Phys. Chem. B.

[71] Xia M (2019). Photoluminescence properties and energy transfer in a novel Sr_8_ZnY(PO_4_)_7_: Tb^3+^, Eu^3+^ phosphor with high thermal stability and its great potential for application in warm white light emitting diodes. J. Mater. Chem. C.

[72] Yu ZM (2019). Differentiation of photon generation depended on electrospun configuration in Eu^3+^/Tb^3+^ doped polyacrylonitrile nanofibers. J. Alloys Compd..

[73] Littlefair PJ (1985). The luminous efficacy of daylight: a review. Lighting Res. Technol..

